# The association between inflammatory and immune system biomarkers and the dietary inflammatory index in patients with COVID-19

**DOI:** 10.3389/fnut.2023.1075061

**Published:** 2023-03-31

**Authors:** Somayyeh Barania Adabi, Sevana Daneghian, Hamidreaza Khalkhali, Rahim Nejadrahim, Nitin Shivappa

**Affiliations:** ^1^Maternal and Childhood Obesity Research Center, Urmia University of Medical Sciences, Urmia, Iran; ^2^Department of Nutrition, School of Medicine, Urmia University of Medical Sciences, Urmia, Iran; ^3^Food and Beverages Safety Research Center, Urmia University of Medical Sciences, Urmia, Iran; ^4^School of Public Health, Urmia University of Medical Sciences, Urmia, Iran; ^5^Department of Infectious Diseases and Dermatology, Urmia University of Medical Sciences, Urmia, Iran; ^6^Cancer Prevention and Control Program, Department of Epidemiology and Biostatistics, Arnold School of Public Health, University of South Carolina, Columbia, SC, United States

**Keywords:** dietary inflammatory index, inflammation, immune system, hospitalization, COVID-19

## Abstract

**Background:**

Inflammation and cytokine storm have been reported to be the main cause of acute symptoms of coronavirus disease (COVID-19). Diet-induced inflammation may affect the condition of patients with COVID-19. Therefore, this study aimed to investigate the relationship between disease severity, inflammatory and immune system biomarkers, and the dietary inflammatory index (DII) in patients with COVID-19.

**Methods:**

This cross-sectional study was conducted on 500 adult patients with COVID-19. Patients were divided into mild, moderate, and severe conditions based on clinical and laboratory evidence. A validated food frequency questionnaire (FFQ) was used to determine DII and energy-adjusted DII (E-DII) scores. The serum C-reactive protein (CRP) level and blood cell count were measured for all patients. Multiple linear regression was used to explore the association between DII and E-DII and CRP, blood cell counts, and hospitalization in patients with COVID-19.

**Results:**

Coronavirus disease (COVID-19) patients with higher DII had higher consumption of fat and carbohydrate and lower intakes of protein, anti-inflammatory nutrients, garlic, caffeine, tea, onion, and fiber (*P* < 0.05). There was a positive association between DII and CRP (β = 1.024, *P* < 0.001), hospitalization (β = 1.062, *P* < 0.001), WBC count (β = 0.486, *P* < 0.009), neutrophil count (β = 0.565, *P* < 0.001), and neutrophil-to-lymphocyte ratio (β = 0.538, *P* < 0.001) and a negative association between DII and the lymphocyte count (β = −0.569, *P* < 0.001). There was a positive association between E-DII and hospitalization (β = 1.645, *P* < 0.001), WBC count (β = 0.417, *P* < 0.02), and neutrophil-to-lymphocyte ratio (β = 0.35, *P* < 0.03).

**Conclusion:**

There is a positive correlation between DII and inflammation, immune hyperactivation, and length of hospital stay in patients with COVID-19. Further longitudinal studies are necessary.

## Introduction

Since 2019, the world has been grappling with a major health crisis caused by severe acute respiratory syndrome virus 2 (SARS-CoV-2). To date, ~579 million confirmed cases of COVID-19 and 6.4 million deaths have been reported worldwide (1). A COVID-19 infection can present as an asymptomatic infection, or patients can present with a mild upper respiratory tract illness that includes cough, chills, fever, fatigue, and shortness of breath. In severe cases, the most common complications are acute respiratory distress syndrome (ARDS), heart failure, and septic shock. However, severe viral pneumonia with respiratory failure can potentially lead to death (2). Multiple organ dysfunction is probably caused by uncontrolled acute inflammation due to cytokine storm (3).

It has been reported that the severity of the disease is related to socioeconomic status, demographic factors such as age (age over 60 years) and male gender, having an underlying disease, time of initiation of treatment, quality of health care (4), ethnicity (black and South Asian) (5, 6), nutritional and lifestyle items (such as diet, lack of vitamin D level, and obesity) (7–9), smoking (10), and viral load (11).

Many studies have shown that inflammatory status is associated with dietary patterns in patients with COVID-19 (12–16). Adequate intake of fruits, vegetables, olive oil, nuts, and whole grains, which are components of the Mediterranean diet, is negatively associated with inflammation in patients with COVID-19 (12, 17, 18). Moreover, some of the nutrients including vitamins D and A, zinc, selenium, flavonoids, and unsaturated fatty acids can reverse inflammatory conditions in these patients, especially by affecting nuclear factor kappa-B (NF-κB), which is the main production regulator of pro-inflammatory cytokines such as interleukin-6 (IL-6) and tumor necrosis factor-alpha (TNF-α). However, foods with higher glycemic load, simple carbohydrates, saturated fatty acids, processed foods, and red meats found in Western diets are positively associated with inflammation in COVID-19 (19–22).

The dietary inflammatory index (DII) was designed to assess the inflammatory potential of the diet by Shivapa et al. The DII scores the diet based on the intake of 45 foods that affect the concentration of certain inflammatory biomarkers such as IL-6, TNF-α, and C-reactive protein (CRP) or anti-inflammatory biomarkers such as IL-10 and IL-4 (23–26). Recently, studies have shown that DII had a positive relationship with the amount of fibrinogen and white blood cells (lymphocyte, monocyte, neutrophil, neutrophil-to-lymphocyte ratio, eosinophil, and basophil) (24, 27).

To the best of our knowledge, only Moludi et al. have reported that energy-adjusted DII (E-DII) was related to an increased risk of developing COVID-19, as well as CRP and severity of the disease. However, their sample size was low, and studied biomarkers were limited (13). As a result, the present study was conducted to determine the relationship between DII and E-DII, disease severity, and inflammatory and immune system biomarkers in patients with COVID-19.

## Methods

### Study population

The study population of the present consecutive cross-sectional study was adult patients with COVID-19 who were referred to Taleghani Hospital in Urmia, Iran. Based on convenience sampling, all patients with COVID-19 diagnosed with clinical symptoms and quantitative polymerase chain reaction (qPCR) testing from March 2021 to September 2021 were included in the study (28). Patients with obesity (BMI ≥ 30) (29), pregnancy (30), and surgery (31) were excluded. The sample size was calculated based on the study of Shivappa et al. (32) with the correlation coefficient *r* = 0.12. Taking into account the confidence interval of 95% and the study power of 80%, the sample size of 462 was calculated. Considering a 10% dropout rate, the sample size was calculated as 500 patients.

After the diagnosis of COVID-19, the patients were divided into inpatient and outpatient groups based on evidence such as arterial oxygen saturation, respiratory rate, clinical symptoms, and lung involvement in CT scans. The criteria for dividing individuals into mild, moderate, severe, and critical [intensive care unit (ICU)] categories have been previously reported (2).

The Ethics Committee of Urmia University of Medical Sciences, Urmia, Iran, approved the study protocol (ethics code IR.UMSU.REC.1399.367). This research complies with the standards of the Declaration of Helsinki and current ethical guidelines.

### Dietary inflammatory index

Regarding the assessment of dietary intake, the validated 147-item food frequency questionnaire (FFQ) (33) was filled out by phone call, based on the recommendations of the ethics committee, which did not allow direct contact between the researchers and the patients with COVID-19. Food intake analysis was then performed after converting all units to grams using Nutritionist-IV software (version 7.0; NSquared computing, Salem, OR, USA) to check the intake of the year before the disease. Before filling out the questionnaire, images containing food units were sent to the patients' mobile phones through applications or e-mail. After the full explanation of the food units, a phone call was made to the patient and the questionnaire was completed.

After completing the FFQ, Shivappa et al.'s method (34) was used to determine DII scores. They reported a total of 45 specific nutrients and food items that affect the concentration of some inflammatory biomarkers such as interleukin-6 (IL-6), tumor necrosis factor-alpha (TNF-α), IL-1β, and C-reactive protein (CRP) or anti-inflammatory biomarkers such as IL-10 and IL-4. Accordingly, the inflammatory potential of each food was evaluated according to either the increase in inflammatory factors, the decrease in anti-inflammatory factors, or the lack of effect on inflammation. In the first step, the mean and standard deviation for each of the 45 food parameters were estimated based on data that were then linked to a regionally representative global database. A z-score was calculated by subtracting the “standard report mean” from the “standard global mean” and then dividing it by its standard deviation. The calculated value is converted to a percentile score to minimize the effect of ‘right skewing’, and then, this value is multiplied by the respective inflammatory effect score to derive the overall DII score. A total of 28 nutritional parameters including energy, carbohydrates, total fat, protein, saturated fat, cholesterol, polyunsaturated fatty acids (PUFAs), monounsaturated fatty acids (MUFAs), fiber, vitamins A, C, D, and E, beta-carotene, B vitamins (B1, B2, B3, B6, B9, and B12), iron, magnesium, zinc, selenium, garlic, caffeine, tea, and onion were available for the current FFQ to calculate DII. Energy-adjusted DII (E-DII) was calculated by derived DII per 1,000 kcal.

### Biochemical measures

According to the protocol for the management of COVID-19, after referring to the hospital and confirming the inclusion criteria, the serum CRP level and blood cell count were performed for all patients.

### Statistical analysis

In this study, data analysis was performed by SPSS software (version 25, Chicago, IL, USA). A normality test was done using the Kolmogorov–Smirnov test and descriptive data (mean and SD, skewness, kurtosis) (35). Fisher's exact test analyzed the distribution of qualitative variables among the DII and E-DII quartiles and different stages of COVID-19. A one-way analysis of variance (ANOVA) (the Kruskal–Wallis test for non-normally distributed data) was used to determine the difference in quantitative variables among the DIIs and E-DII quartiles. *Post-hoc* analyses were performed based on Tukey's test. A correlation test was performed to investigate the relationship between the severity of the disease, inflammatory and immune system biomarkers, and the DII and E-DII values. Multiple linear regression was used to explore the association between DII and E-DII and CRP, blood cell counts, and hospitalization in patients with COVID-19 after adjusting for age, gender, literacy level, job, economic level, smoking, dietary supplement, drug history, disease history, BMI, and physical activity. In the cases of skewed distribution, log transformation was performed. The regression assumptions including residual normality, residual independence, homogeneity of residual variances, and co-linearity were confirmed by performing a normal probability plot, Durbin-Watson statistics (1.5–2.5), residual vs. predicted value plot, and variance inflation factor (VIF < 5). *P*-values <0.05 were considered statistically significant in all analyses.

## Results

### Baseline characteristics of patients

**Figure 1:**
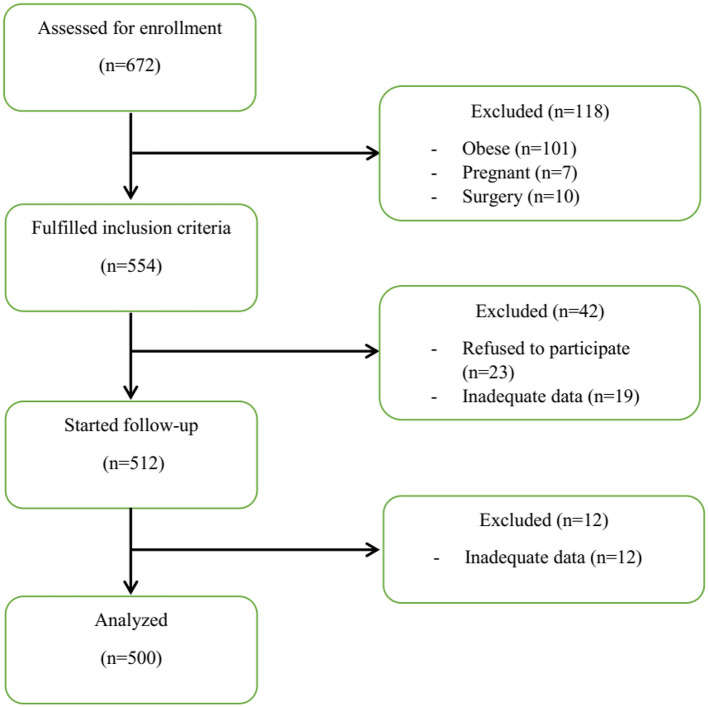
Overview of the study.

The flowchart of the study process is shown in Figure 1. Finally, 500 patients were examined. As shown in Table 1, patients in the severe and ICU groups had higher age, weight, and body mass index (BMI) compared to the moderate and mild groups (*P* < 0.05). Moreover, the grouping of patients based on the DII and E-DII quartiles showed that patients with increasing age (mean age 40.33 years in quartile I vs. 45.2 years in quartile IV), weight (mean weight 72.27 kg in quartile I vs. 75.44 kg in quartile IV), and BMI (mean BMI 24.85 kg/m^2^ in quartile I vs. 26.22 kg/m^2^ in quartile IV) were placed in a higher subgroup of the DII quartile (*P* < 0.05). There was a similar trend for the E-DII quartiles (*P* < 0.05). There were no significant differences between patients with different disease severity in terms of gender and smoking status (Table 1). These findings were shown for patients in different quartiles of DII and E-DII (*P* > 0.05). As reported in Table 1, 25.5% of ICU patients with COVID-19 had a medical history such as heart disease, hypertension, diabetes mellitus, fatty liver disease, hypothyroidism, anemia, asthma, and depression; however, this rate was 13.8% in mild patients (*P* < 0.05). This trend was similar in DII (*P* = 0.003) and E-DII quartiles (*P* = 0.001); therefore, patients in the highest quartiles of DII (29.6% vs. 11.2%) and E-DII (32% vs. 11.2%) had the highest rate of disease history compared to the lowest quartiles. Moreover, ICU patients with COVID-19 had the highest rate of medication use (22.4% vs. 11.3%) compared to mild patients (Table 1). In the DII and E-DII quartiles, there were no significant differences in terms of dietary supplement history (*P* > 0.05).

**Table 1:** Comparison of baseline characteristics between different stages of COVID-19 (*N*_total_ = 500).

**Variable**	**Mild**	**Moderate**	**Severe**	**ICU**	***P*-value**
Age (year)^*^	40.96 ± 11.19	41.08 ± 10.21	43.35 ± 10.45	46.83 ± 10.01	0.001
Weight (kg)^*^	71.24 ± 9.58	75.88 ± 8.99	76.36 ± 9.19	79.92 ± 7.93	<0.001
BMI (kg/m^2^)^*^	24.55 ± 1.28	25.69 ± 2.17	25.97 ± 2.11	27.3 ± 1.74	<0.001
Gender^#^					0.912
Men	40 (50)	110 (48)	70 (51.9)	30 (54.5)	
Women	40 (50)	120 (52)	65 (48.1)	25 (45.5)	
Smoking^#^					0.467
Yes	8 (10)	18 (7.8)	6 (4.4)	4 (7.3)	
No	72 (90)	212 (92.2)	129 (95.6)	51 (92.7)	
Disease history^#^					<0.001
Yes	11 (13.8)	32 (13.9)	46 (34.1)	14 (25.5)	
No	69 (86.3)	198 (86.1)	89 (65.9)	41 (74.5)	
Drug history^#^					<0.001
Yes	9 (11.3)	28 (12.2)	38 (28.4)	13 (22.4)	
No	71 (88.8)	202 (87.8)	96 (71.6)	42 (77.6)	
Supplement history^#^					0.004
Yes	25 (31.3)	36 (15.7)	37 (27.4)	8 (14.5)	
No	55 (68.7)	194 (84.3)	98 (72.6)	47 (85.5)	

BMI, body mass index; ICU, intensive care unit.

* Comparison of quantitative variables using one-way ANOVA (presented as mean ± SD).

# Comparison of qualitative variables using the Chi-square test [presented as frequency (within group percentage)].

### Relationship between biochemical variables, dietary factors, and disease severity, and DII and E-DII

As reported in Tables 2, 3, patients with COVID-19 in quartile IV of DII and E-DII significantly had higher CRP, neutrophil percent, and NLR and lower lymphocyte percent (*P* < 0.05). For WBC, a significant difference was shown only in the DII quartiles. *Post-hoc* analyses based on Tukey's test showed that all subgroups had significant differences two by two.

**Table 2:** Biochemical measures, dietary factors, and disease severity among patients with different DII quartiles.

**Variable**	**DII quartile**				***P*-value**
	**I**	**II**	**III**	**IV**	
CRP^*^ (mg/l)	6.24 ± 4.93	21.46 ± 12.33	42.49 ± 12.62	59.91 ± 13.79	<0.001
WBC^#^ (count)	5,800 (4,300–8,050)	5,500 (4,000–7,600)	6,200 (4,900–8,100)	6,600 (4,700–9,650)	0.006
Lymphocyte^#^ (%)	30 (22–3.7)	23 (15–30)	17 (11–24)	14 (10–20)	<0.001
Neutrophil^*^ (%)	65.53 ± 11.64	72.4 ± 10.82	78.15 ± 10.93	82.08 ± 7.53	<0.001
NLR^#^	2.13 (1.63–3.25)	3.17 (2.18–5.16)	4.88 (3.02–8)	5.71 (3.8–9)	<0.001
Blood pressure^*^ (mmHg)	114.76 ± 10.02	115.69 ± 10.81	114.36 ± 10.26	117.74 ± 12.64	0.072
Energy^*^ (kcal/d)	2,242.49 ± 203.8	2,348.83 ± 308.77	2,359.51 ± 335/98	2,524.1 ± 324.02	<0.001
Protein^*^ (g/d)	85.02 ± 8.03	81.51 ± 10.11	78.79 ± 9.25	78.36 ± 9.01	<0.001
Carbohydrate^*^ (g/d)	305.89 ± 36.21	325.69 ± 53.59	326.62 ± 58.24	345.77 ± 55.45	<0.001
Fat^*^ (g/d)	73.43 ± 8.34	81.21 ± 12.75	84.83 ± 15.57	92.69 ± 13.71	<0.001
Fiber^#^ (g/d)	29.56 (27.66–32.99)	29.43 (26.93–31.84)	28.61 (26.18–31.34)	28.48 (26.2–31.3)	0.014
MUFAs^*^ (g/d)	24.42 ± 2.74	25.28 ± 4.1	26.09 ± 4.75	25.89 ± 4.38	0.006
PUFAs^*^ (g/d)	15.15 ± 2.47	13.49 ± 1.97	12.24 ± 2.04	11.68 ± 1.68	<0.001
SFAs^*^ (g/d)	24.91 ± 5.16	32 ± 8.64	35.98 ± 10.99	44.05 ± 11.62	<0.001
Cholesterol^*^ (mg/d)	294.72 ± 64.81	353.54 ± 103.13	367.9 ± 109.75	391.3 ± 101.47	<0.001
Vitamin A^*^ (mcg RAE/d)	847.89 ± 233.65	823.67 ± 196	819.02 ± 213.68	779.4 ± 167.98	0.066
Beta carotene^*^ (mcg/d)	3,044.89 ± 645.4	2,362.48 ± 544.9	1,934.3 ± 414.39	1,660.03 ± 367.7	<0.001
Vitamin C^*^ (mg/d)	152.66 ± 25.4	136.21 ± 27.02	125.14 ± 32.98	104.56 ± 25.02	<0.001
Vitamin D^#^ (mcg/d)	1.68 (1.43–1.93)	1.68 (1.32–1.98)	1.5 (1.24–1.84)	1.34 (1.18–1.56)	<0.001
Vitamin E^#^ (mg/d)	8.13 (7.17–8.91)	7.05 (6.17–7.98)	6.24 (5.4–6.98)	5.89 (5.15–6.36)	<0.001
Vitamin B1^*^ (mg/d)	1.65 ± 0.24	1.54 ± 0.20	1.48 ± 0.22	1.51 ± 0.23	<0.001
Vitamin B2^*^ (mg/d)	1.57 ± 0.28	1.33 ± 0.22	1.26 ± 0.22	1.22 ± 0.20	<0.001
Vitamin B3^*^ (mg/d)	27.38 ± 12.20	19.42 ± 5.52	17.74 ± 4.73	16.63 ± 2.6	<0.001
Vitamin B6^*^ (mg/d)	1.80 ± 0.51	1.67 ± 0.16	1.57 ± 0.16	1.54 ± 0.15	<0.001
Vitamin B9^*^ (mcg/d)	314.81 ± 166.83	263.26 ± 114.82	240.54 ± 43.97	231.36 ± 46.49	<0.001
Vitamin B12^*^ (mcg/d)	2.06 ± 0.59	2.08 ± 0.67	2.17 ± 0.76	2.31 ± 0.65	0.014
Iron^*^ (mg/d)	15.88 ± 2.29	13.8 ± 1.82	13.18 ± 1.91	13.27 ± 1.79	<0.001
Selenium^*^ (mcg/d)	127.31 ± 27.01	123.7 ± 28.49	122.28 ± 23.65	121.97 ± 18.23	0.297
Magnesium^#^ (mg/d)	442.9 (383.2–524.7)	340.5 (314.7–372.5)	301.8 (281–331.2)	282.7 (266.3–302)	<0.001
Zinc^#^ (mg/d)	9.44 (8.88–10.24)	8.47 (7.67–9.05)	7.94 (7.39–8.64)	7.77 (7.07–8.3)	<0.001
Garlic^#^ (g/d)	1.28 (0.85–2.14)	0.85 (0.6–1.28)	0.85 (0.4–1.28)	0.85 (0.42–1)	<0.001
Caffeine^*^ (mg/d)	183.77 ± 50.78	179.31 ± 56.14	164.78 ± 55.05	159.88 ± 58.89	0.001
Tea^*^ (g/d)	838.25 ± 225.55	865.23 ± 260.49	790.06 ± 292.7	769.28 ± 280.93	0.018
Onion^*^ (g/d)	21.78 ± 5.39	18.91 ± 5.42	17.2 ± 5.96	14.84 ± 5.18	<0.001
Disease severity^%^ (*n*/%)					<0.001
Mild	67 (53.6)	11 (8.8)	2 (1.6)	0 (0)	
Moderate	58 (46.4)	88 (70.4)	66 (52.8)	18 (14.4)	
Severe	0 (0)	23 (18.4)	42 (33.6)	70 (56)	
ICU	0 (0)	3 (2.4)	15 (12)	37 (29.6)	
Hospitalization^#^ (day)	1 (1–5)	5 (5,6)	6 (5–7)	7 (5–10)	<0.001

CRP, C-reactive protein; WBC, white blood cell; NLR, neutrophil-to-lymphocyte ratio; MUFAs, monounsaturated fatty acids; PUFAs, polyunsaturated fatty acids; SFA, saturated fatty acids; ICU, intensive care unit.

* Presented as mean ± SD and analyzed by one-way ANOVA.

# Presented as median (IQR) and analyzed by the Kruskal–Wallis test.

% Analyzed by the chi-square test.

**Table 3:** Biochemical measures, dietary factors, and disease severity among the energy-adjusted dietary inflammatory index (E-DII) quartiles.

**Variable**	**E-DII quartile**				***P*-value**
	**I**	**II**	**III**	**IV**	
CRP^*^ (mg/l)	6.49 ± 3.31	22.28 ± 13.92	43.01 ± 13.74	58.31 ± 14.86	<0.001
WBC^#^ (count)	5,800 (4,350–8,050)	5,500 (4,050–8,000)	6,100 (4,600–8,950)	6,600 (5,000–8,700)	0.09
Lymphocyte^#^ (%)	30 (23–37)	21 (14–29.5)	15 (10.5–23.5)	15 (10–21)	<0.001
Neutrophil^*^ (%)	65.45 ± 11.55	74.29 ± 10.16	78.25 ± 12.37	80.16 ± 8.18	<0.001
NLR^#^	2.13 (1.63–3.17)	3.5 (2.2–5.71)	5.46 (2.99–8.25)	5.18 (3.51–8.5)	<0.001
Blood pressure^*^ (mmHg)	114.93 ± 10.16	115.95 ± 10.98	115.52 ± 11.38	116.14 ± 11.62	0.83
Energy^*^ (kcal/d)	2,255.49 ± 222.31	2,443.8 ± 319.26	2,474.8 ± 341.59	2,300.86 ± 304.92	<0.001
Protein^*^ (g/d)	85.17 ± 8.03	83.67 ± 9.82	81.02 ± 8.87	73.82 ± 6.76	<0.001
Carbohydrate^*^ (g/d)	307.23 ± 37.57	343.47 ± 57.75	340.2 ± 56.61	313.08 ± 49.65	<0.001
Fat^*^ (g/d)	73.87 ± 9.47	84.78 ± 13.86	88.21 ± 15.87	85.31 ± 14.24	<0.001
Fiber^#^ (g/d)	29.71 (27.72–33.03)	30 (27.13–32.64)	29.97 (27.87–31.97)	26.9 (24.86–28.78)	<0.001
MUFAs^*^ (g/d)	24.42 ± 2.73	26.28 ± 4.55	25.42 ± 4.39	25.55 ± 4.3	0.004
PUFAs^*^ (g/d)	15.18 ± 2.46	13.7 ± 1.86	12.26 ± 1.84	11.43 ± 1.73	<0.001
SFAs^*^ (g/d)	25.2 ± 6.31	34.01 ± 9.69	39.42 ± 12.94	38.23 ± 11.08	<0.001
Cholesterol^*^ (mg/d)	297.18 ± 70.79	366.13 ± 100.4	378.1 ± 115.26	366.05 ± 99.03	<0.001
Vitamin A^*^ (mcg RAE/d)	844.2 ± 188.34	822.45 ± 218.97	818.54 ± 226.16	784.79 ± 181.46	0.146
Beta carotene^*^ (mcg/d)	3,037.32 ± 654.4	2,295.26 ± 548.42	1,911.12 ± 498.7	1,758.01 ± 726.08	<0.001
Vitamin C^*^ (mg/d)	152.98 ± 25.5	135.18 ± 26.27	122.44 ± 31.47	107.98 ± 29.75	<0.001
Vitamin D^#^ (mcg/d)	1.65 (1.38–1.83)	1.65 (1.35–2.02)	1.55 (1.3–1.94)	1.36 (1.18–1.6)	<0.001
Vitamin E^#^ (mg/d)	8.05 (7.13–8.91)	7.3 (6.27–8.22)	6.21 (5.56–6.79)	5.65 (5.06–6.37)	<0.001
Vitamin B1^*^ (mg/d)	1.66 ± 0.24	1.57 ± 0.20	1.54 ± 0.22	1.41 ± 0.19	<0.001
Vitamin B2^*^ (mg/d)	1.58 ± 0.28	1.33 ± 0.21	1.29 ± 0.22	1.12 ± 20	<0.001
Vitamin B3^*^ (mg/d)	27.26 ± 12.27	19.84 ± 5.53	18.12 ± 4.49	15.96 ± 2.43	<0.001
Vitamin B6^*^ (mg/d)	1.81 ± 0.16	1.67 ± 0.17	1.62 ± 0.14	1.5 ± 0.14	<0.001
Vitamin B9^*^ (mcg/d)	315.4 ± 165.95	265.11 ± 115.72	240.07 ± 39.16	229.39 ± 49.27	<0.001
Vitamin B12^*^ (mcg/d)	2.09 ± 0.62	2.16 ± 0.70	2.19 ± 0.70	2.18 ± 0.67	0.66
Iron^*^ (mg/d)	15.89 ± 2.29	14.06 ± 1.82	13.81 ± 1.69	12.37 ± 1.58	<0.001
Selenium^*^ (mcg/d)	129.86 ± 26.46	129.05 ± 28.41	122.14 ± 18.71	114.21 ± 20.81	<0.001
Magnesium^#^ (mg/d)	442.9 (372.6–524.7)	341.1 (317.8–387.9)	305.3 (292.1–337.3)	272.6 (258–290)	<0.001
Zinc^#^ (mg/d)	9.43 (8.95–10.23)	8.57 (7.88–9.11)	8.23 (7.63–8.65)	7.43 (6.95–7.97)	<0.001
Garlic^#^ (g/d)	1.28 (0.85–2.14)	0.85 (0.6–1.28)	0.85 (0.42–1)	0.85 (0.42–1)	<0.001
Caffeine^*^ (mg/d)	184.63 ± 52.2	169.81 ± 54.89	168.81 ± 56.78	164.58 ± 58.56	0.027
Tea^*^ (g/d)	842.32 ± 234.7	824.62 ± 282.16	809 ± 276.14	786.89 ± 276.64	0.409
Onion^*^ (g/d)	21.96 ± 5.4	18.14 ± 5.42	17.42 ± 5.98	15.18 ± 5.31	<0.001
Disease severity^%^ (*n*/%)					<0.001
Mild	68 (54.4)	10 (8)	2 (1.6)	0 (0)	
Moderate	56 (44.8)	78 (62.4)	54 (43.2)	42 (33.6)	
Severe	1 (0.8)	29 (23.2)	48 (38.4)	57 (45.6)	
ICU	0 (0)	8 (6.4)	21 (16.8)	26 (20.8)	
Hospitalization^#^ (day)	1 (1–5)	5 (5,6)	6 (5–8)	6 (5–9)	<0.001

CRP, C-reactive protein; WBC, white blood cell; NLR, neutrophil-to-lymphocyte ratio; MUFAs, monounsaturated fatty acids; PUFAs, polyunsaturated fatty acids; SFA, saturated fatty acids; ICU, intensive care unit.

* Presented as mean ± SD and analyzed by one-way ANOVA.

# Presented as median (IQR) and analyzed by the Kruskal–Wallis test.

% Analyzed by the Chi-square test.

In terms of macronutrients and energy, patients with COVID-19 in quartile IV of DII had the highest consumption of energy, fat, and carbohydrate and the lowest protein intake (*P* < 0.05) (Table 2). *Post hoc* analysis revealed that there were no significant differences between quartiles 2 and 3 in terms of energy and macronutrients. Despite the significant difference, there is no clear trend for energy and macronutrient values in the E-DII quartiles (Table 3). However, by decreasing the consumption of anti-inflammatory nutrients (vitamin D, vitamin E, vitamin A, vitamin C, beta-carotene, magnesium, zinc, selenium, PUFAs, B1, B2, B3, and B6), garlic, caffeine, tea, onion, and fiber, the DII and E-DII values increased (Tables 2, 3). Vitamin B12 intake was not different among the E-DII quartiles (*P* = 0.66) (Table 3). However, with the increase in the DII quartiles, B12 intake increased significantly (*P* = 0.014) (Table 2). Iron and vitamin B9 intakes were significantly higher in the first DII and E-DII quartiles (Table 2). However, *post-hoc* analysis revealed that iron and vitamin B9 intakes in other quartiles of DII were not significantly different. The median day of hospitalization in quartile IV of DII and E-DII was 7 and 6 days, respectively. However, the median day of hospitalization in the first quartile of DII and E-DII was only one day (*P* < 0.05) (Tables 2, 3). Most of the patients with mild and moderate COVID-19 had low DII and E-DII. However, ICU patients and patients with severe COVID-19 consumed a diet with higher DII and E-DII (*P* < 0.05) (Tables 2, 3).

Based on correlation tests, significant positive correlations were found between CRP, neutrophil, WBC, NLR, hospitalization, BMI, weight, B12, MUFAs, SFA, energy, cholesterol, fat, carbohydrate intakes, and DII values. However, significant negative correlations were reported between DII values and lymphocyte, intakes of anti-inflammatory nutrients, iron, protein, garlic, caffeine, onion, and fiber intakes (*P* < 0.05) (Supplementary Table 1). Significant relationships regarding vitamin A, vitamin B12, WBC, carbohydrates, and MUFAs disappeared following energy adjustment for DII (Supplementary Table 2).

The results of the multiple linear regression found a positive association between DII and CRP (β = 1.024, *P* < 0.001), hospitalization (β = 1.062, *P* < 0.001), WBC count (β = 0.486, *P* < 0.009), neutrophil count (β = 0.565, *P* < 0.001), and neutrophil-to-lymphocyte ratio (β = 0.538, *P* < 0.001) and a negative association between DII and the lymphocyte count (β = −0.569, *P* < 0.001) in patients with COVID-19 after adjusting for age, gender, literacy level, job, economic level, smoking, dietary supplement, drug history, disease history, BMI, and physical activity (Table 4). Regarding the association of E-DII with inflammatory and immune system biomarkers, a positive association was found between E-DII and hospitalization (β = 1.645, *P* < 0.001), WBC count (β = 0.417, *P* < 0.02), and neutrophil-to-lymphocyte ratio (β = 0.35, *P* < 0.03) in patients with COVID-19 after adjusting for age, gender, literacy level, job, economic level, smoking, dietary supplement, drug history, disease history, BMI, and physical activity (Table 5).

**Table 4:** Association of inflammatory and immune system biomarkers with dietary inflammatory index (DII) in patients with COVID-19 based on multiple linear regression.

**Variables**	**Unstandardized beta (B)**	**SE**	**Standardized beta**	*P*-value^*^
CRP	22.25	1.95	1.024	<0.001
Hospitalization	4.879	0.784	1.062	<0.001
WBC count	0.12	0.046	0.486	0.009
Neutrophil count	6.636	1.855	0.565	<0.001
Lymphocyte count	−6.273	1.76	−0.569	<0.001
Neutrophil-to-lymphocyte ratio	2.771	0.86	0.538	0.001

CRP, C-reactive protein.

* Adjusted for age, gender, literacy level, job, economic level, smoking, dietary supplement, drug history, disease history, BMI, and MET.

**Table 5:** Association of inflammatory and immune system biomarkers with energy-adjusted dietary inflammatory index (E-DII) in patients with COVID-19 based on multiple linear regression.

**Variables**	**Unstandardized beta (B)**	**SE**	**Standardized beta**	*P*-value^*^
CRP	7.691	4.485	0.149	0.087
Hospitalization	7.049	1.632	0.645	< 0.001
WBC count	0.245	0.105	0.417	0.02
Neutrophil count	4.499	4.259	0.161	0.291
Lymphocyte count	−4.754	4.04	−0.181	0.24
Neutrophil-to-lymphocyte ratio	4.291	1.973	0.35	0.03

CRP, C-reactive protein.

* Adjusted for age, gender, literacy level, job, economic level, smoking, dietary supplement, drug history, disease history, BMI, and MET.

## Discussion

This study aimed to evaluate the association between DII and inflammatory biomarkers, immune system biomarkers, and disease severity in patients with COVID-19. Our results showed that there were positive significant relationships between DII and CRP, hospitalization, neutrophil, WBC, NLR, and disease severity. Similar to our results, various observational studies have reported that higher DII increases the risk of lung-related inflammatory conditions such as asthma (36, 37) and COVID-19 (13, 38). Similarly, studies conducted on other health conditions have reported positive associations between CRP and immune system biomarkers with DII values (39, 40). In terms of hospitalization, DII and E-DII values were positively related to hospitalization. In line with our findings, DII was a determinant of hospitalization length in other diseases (41, 42).

In a mechanistic review study conducted by Maiorino et al. (43), the proposed mechanisms of the beneficial effect of the Mediterranean dietary pattern (including whole grains, vegetables and fruits, fish, and “healthy” fats, including MUFA and PUFA) for controlling COVID-19 were inhibition of inflammation caused by COVID-19, characterized by increased CRP, TNF-α, and interleukin-6, as well as infection through supplying of vitamin D, which reduces the expression of angiotensin-converting enzyme 2 (ACE2) receptor and thus decreases the entry of pathogens into lung cells. Finally, it was suggested that prospective studies must be conducted to prove the beneficial effect of the Mediterranean dietary pattern on the control of COVID-19 infection in large populations (43). Butler et al. suggested that following a Western diet that was associated with more consumption of red meats, saturated fats, and refined sugars leads to activation of the innate immune system and impairment of acquired immunity, subsequently leading to chronic inflammation and disruption of the host's defense against viruses (44).

Our results showed that higher intakes of anti-inflammatory micro-nutrients (vitamins A, D, and E as fat-soluble vitamins, vitamins C, B1, B2, B3, B6, and B9 as water-soluble vitamins, beta-carotene, iron, magnesium, zinc, and selenium) led to the lower DII and E-DII values. Similarly, Özbey et al. reported that intakes of vitamins A, E, and C had a negative relationship with DII values (36). Kim et al. also demonstrated that dietary vitamins A, D, E, C, B1, B2, B3, B6, and B9, and beta-carotene, zinc, selenium, and magnesium were negatively associated with DII values (45).

Different studies have investigated the anti-inflammatory mechanisms of vitamin D (46), vitamin E (47), vitamin A (48), vitamin C (49), beta-carotene (50), magnesium (51), zinc (52), selenium (53), and B vitamins (54). However, an overload of some minerals such as iron leads to inflammatory responses (55). Some anti-inflammatory features have been proposed for vitamin B12 in COVID-19 (56). An insignificant relationship between E-DII and vitamin B12 in our analysis showed that a significant positive association between dietary vitamin B12 and DII might be biased and an energy-adjusted correlation could fix this bias. Moreover, there was a positive correlation between the CRP level and the B12 level in the first 2 days of ICU admission (57). Therefore, additional studies must be done to clear the association between B12 and inflammation in patients with COVID-19.

In terms of macronutrients, negative relationships between protein, PUFA, and fiber intakes with DII and E-DII values were shown in our study. However, energy, SFA, cholesterol, fat, and carbohydrate intakes resulted in higher DII and E-DII values. Dietary intake of MUFAs was only positively related to DII and not E-DII. In a study by Aghababayan et al., only dietary intakes of total fat, cholesterol, and SFA were significantly higher in the upper tertile of DII (58). In another study, dietary intakes of carbohydrates, fibers, and PUFAs were higher in the lowest tertile of DII; however, dietary intakes of cholesterol, SFAs, and MUFAs were higher in the third quartile of DII (59).

The anti-inflammatory effects of PUFAs have been described in another study (60). Short-chain fatty acids (SCFAs) produced by microflora in the intestinal duct are the main contributors to reduced inflammation following fiber intake (61). In a large cohort study, higher protein intake, particularly from plant-based foods, was inversely associated with inflammation (62). However, Koelman et al. reported that both high-protein and low-protein diets lead to a decrease in the CRP level. However, this decrease in the high-protein group was more pronounced (63). High dietary methionine content was reported to be associated with higher inflammation (64, 65). Most of the studies that addressed the positive effect of high-protein intake on inflammation have pointed to the high consumption of protein from plant or dairy sources (66, 67). However, the positive effect of dairy products may be related to the bacterial content of fermented dairy products or bioactive peptides (68). Various studies have reported the beneficial effect of MUFAs on inflammation (69, 70). The positive association between MUFAs and DII values in the current study was lost by adjusting the effect of energy. As a result, the positive relationship between MUFAs and DII could be mainly due to the confounding effect of energy consumption on MUFA intake. Numerous studies have demonstrated that SFAs induce adipose tissue inflammation mediated by toll-like receptors (TLRs) as the main receptors in the innate immune system. It has been suggested that this stimulation is mediated by the SFA effect on TLR receptor numbers and dimerization, the balance between SFAs and USFA, and lipid rafts (71). *In vivo* studies have revealed that high dietary cholesterol leads to increased adipocyte size, expression of pro-inflammatory cytokines, and macrophage infiltration (72, 73). Karimi et al. reported that high carbohydrate intake had a positive association with monocyte chemoattractant protein-1 (MCP-1) and transforming growth factor beta (TGF-β) that were related to increased expression of NF-κB as the main regulator of pro-inflammatory cytokine expression (74).

Our investigation showed that dietary intakes of garlic, onion, caffeine, and tea were also negatively related to DII and E-DII values. These functional foods may have anti-inflammatory effects due to their flavonoid components (75, 76).

It must be noted that the dietary patterns of people during the COVID-19 pandemic were changed due to quarantine rules. In a study by Górnicka et al. in Poland, 34%, 33%, and 18% of respondents reported an increase in total food, sweets, and alcohol consumption, respectively. A total of 53% of respondents did not change their eating habits during the COVID-19 pandemic (77). Sidor et al. reported an increase in total energy intake in people from Poland during the COVID-19 quarantine, which was associated with less consumption of vegetables, fruits, and legumes and a tendency to consume more meat, dairy, and fast foods (78). Hu et al. reported that only 30% of people in China increased vegetable and fruit consumption during the COVID-19 quarantine (79).

Regression analysis was implemented to explore potential predictors of CRP and blood cell counts. Possessing higher DII and E-DII values, being of the male gender, having higher education, and being a smoker were associated with higher CRP in patients with COVID-19. Lee et al. reported that men have higher CRP levels than women (80). However, this trend is ethnically specific and does not generalize to the entire population (81). Consistent with our results, large observational studies also found that socioeconomic status is associated with CRP levels (82–84). We also found that DII, gender, and hospitalization were the common main predictors of blood cell counts. Having higher DII, being of the male gender, and having a longer hospitalization led to higher WBC and neutrophil counts and NLR but a lower lymphocyte count in patients with COVID-19. A long hospitalization period can lead to unwanted acute hospital infections, which affect the immune system and inflammation.

There were some limitations in the present study. First, it would be better to conduct a case–control design to obtain more reliable results. However, healthy individuals to be included in the control group may have been infected with COVID-19, and there was no possibility of performing high-volume PCR testing. Second, only CRP was tested as the main biomarker of systemic inflammation due to the limited budget allocated to the project. Further studies should include other inflammatory biomarkers such as pro-inflammatory cytokines. Third, limitations of the FFQ including the limited structure and response options, and reliance on respondents' memory should be considered (85).

## Conclusion

A positive correlation between DII and E-DII with hyperactivation of the inflammatory and innate immune system and length of hospitalization in patients with COVID-19 was obtained from the present study. However, higher DII and E-DII led to dysfunction in the acquired immune system. The number of patients with severe COVID-19 was higher in the upper DII and E-DII quartiles. More intakes of anti-inflammatory nutrients, protein, garlic, onion, caffeine, tea, and fiber led to lower DII and E-DII values. In contrast, SFA, cholesterol, fat, and carbohydrate intakes led to higher DII and E-DII values. Dietary intake of MUFAs was only positively related to DII and not E-DII.

## Data availability statement

The datasets presented in this article are not readily available because this research is complied with the standards of the Declaration of Helsinki and current ethical guidelines. Requests to access the datasets should be directed to SD, https://orcid.org/0000-0002-6769-1577.

## Ethics statement

The studies involving human participants were reviewed and approved by the Ethics Committee of Urmia University of Medical Sciences, Urmia, Iran (Ethics Code IR.UMSU.REC.1399.367). The patients/participants provided their written informed consent to participate in this study.

## Author contributions

All authors listed have made a substantial, direct, and intellectual contribution to the work and approved it for publication.
