# Supplementary material for: The association between inflammatory and immune system biomarkers and the dietary inflammatory index in patients with COVID-19
Source: Front Nutr. 2023 Mar 31;10:1075061. doi: 10.3389/fnut.2023.1075061 (PMC10103612; doi:10.3389/fnut.2023.1075061)
Supplement: Supplementary file 1 [file Data_Sheet_1.docx]

**Supplementary table 1:** Results of correlation tests between DII and different variables in COVID-19 patients

| **Variables** | **Coefficient, *P*** | **Variables** | **Coefficient, *P*** |
| --- | --- | --- | --- |
| CRP^*^ | 0.865, <0.001 | Vitamin A^*^ | -0.129, 0.004 |
| Neutrophil^*^ | 0.535, <0.001 | Beta carotene^*^ | -0.73, <0.001 |
| WBC^#^ | 0.152, 0.001 | Vitamin C^*^ | -0.555, <0.001 |
| Lymphocyte^#^ | -0.517, <0.001 | Energy | 0.301, <0.001 |
| NLR^#^ | 0.522, <0.001 | Protein^*^ | -0.297, <0.001 |
| Hospitalization^#^ | 0.64, <0.001 | Carbohydrate^*^ | 0.254, <0.001 |
| Blood pressure^*^ | 0.067, 0.138 | Fat^*^ | 0.487, <0.001 |
| Magnesium^#^ | -0.727, <0.001 | Cholesterol^*^ | 0.347, <0.001 |
| Zinc^#^ | -0.579, <0.001 | SFA^*^ | 0.605, <0.001 |
| Selenium^*^ | -0.076, 0.09 | MUFA^*^ | 0.144, 0.001 |
| Iron^*^ | -0.503, <0.001 | PUFA^*^ | -0.58, 0.001 |
| Vitamin B1^*^ | -0.283, <0.001 | Fiber^#^ | -0.157, <0.001 |
| Vitamin B2^*^ | -0.566, <0.001 | Caffeine^*^ | -0.105, 0.019 |
| Vitamin B3^*^ | -0.541, <0.001 | Garlic# | -0.417, <0.001 |
| Vitamin B6^*^ | -0.581, <0.001 | Onion^*^ | -0.438, <0.001 |
| Vitamin B9^*^ | -0.289, <0.001 | Tea^*^ | -0.027, 0.54 |
| Vitamin B12^*^ | 0.92, 0.04 | Weight^*^ | 0.225, <0.001 |
| Vitamin D^#^ | -0.315, <0.001 | BMI^*^ | 0.283, <0.001 |
| Vitamin E^#^ | -0.578, <0.001 | E-DII^*^ | 0.966, <0.001 |

* Pearson correlation coefficient

# Spearman's rank correlation coefficient

**Supplementary table 2:** Results of correlation tests between E-DII and different variables in COVID-19 patients

| **Variables** | **Coefficient, *P*** | **Variables** | **Coefficient, *P*** |
| --- | --- | --- | --- |
| CRP^*^ | 0.823, <0.001 | Vitamin A^*^ | -0.051, 0.25 |
| Neutrophil^*^ | 0.486, <0.001 | Beta carotene^*^ | -0.689, <0.001 |
| WBC^#^ | 0.077, 0.084 | Vitamin C^*^ | -0.533, <0.001 |
| Lymphocyte^#^ | -0.466, <0.001 | Energy | 0.09, 0.045 |
| NLR^#^ | 0.454, <0.001 | Protein^*^ | -0.438, <0.001 |
| Hospitalization^#^ | 0.562, <0.001 | Carbohydrate^*^ | 0.068, 0.13 |
| Blood pressure^*^ | 0.031, 0.486 | Fat^*^ | 0.327, <0.001 |
| Magnesium^#^ | -0.785, <0.001 | Cholesterol^*^ | 0.262, <0.001 |
| Zinc^#^ | -0.686, <0.001 | SFA^*^ | 0.466, <0.001 |
| Selenium^*^ | -0.077, 0.086 | MUFA^*^ | 0.078, 0.08 |
| Iron^*^ | -0.591, <0.001 | PUFA^*^ | -0.62, 0.001 |
| Vitamin B1^*^ | -390, <0.001 | Fiber^#^ | -0.315, <0.001 |
| Vitamin B2^*^ | -0.590, <0.001 | Caffeine^*^ | -0.165, <0.001 |
| Vitamin B3^*^ | -0.566, <0.001 | Garlic# | -0.376, <0.001 |
| Vitamin B6^*^ | -0.638, <0.001 | Onion^*^ | -0.402, <0.001 |
| Vitamin B9^*^ | -0.275, <0.001 | Tea^*^ | -0.088, 0.048 |
| Vitamin B12^*^ | 0.12, 0.792 | Weight^*^ | 0.19, <0.001 |
| Vitamin D^#^ | -0.21, <0.001 | BMI^*^ | 0.255, <0.001 |
| Vitamin E^#^ | -0.6, <0.001 | DII^*^ | 0.966, <0.001 |

* Pearson correlation coefficient

# Spearman's rank correlation coefficient
